# Automated Sella-Turcica Annotation and Mesh Alignment of 3D Stereophotographs for Craniosynostosis Patients Using a PCA-FFNN Based Approach

**DOI:** 10.1097/SCS.0000000000011623

**Published:** 2025-06-27

**Authors:** Freek Bielevelt, Najiba Chargi, Joelle van Aalst, Marloes Nienhuijs, Thomas Maal, Hans Delye, Guido de Jong

**Affiliations:** * Radboudumc 3D Lab, Radboud University Medical Center; † Department of Oral and Maxillofacial Surgery, Radboud University Medical Center Nijmegen, Geert Grooteplein Zuid 10, 6525 GA Nijmegen, The Netherlands; ‡ Department of Pediatric Neurosurgery, AZ Turnhout Campus Sint-Elisabeth, Rubensstraat 166, 2300 Turnhout, België

**Keywords:** 3D stereophotogrammetry, craniosynostosis, feedforward neural network, principal component analysis, sella turcica

## Abstract

**Background:**

Craniosynostosis, characterized by the premature fusion of cranial sutures, can lead to significant neurological and developmental complications, necessitating early diagnosis and precise treatment. Traditional cranial morphologic assessment has relied on CT scans, which expose infants to ionizing radiation. Recently, 3D stereophotogrammetry has emerged as a noninvasive alternative, but accurately aligning 3D photographs within standardized reference frames, such as the Sella–turcica–Nasion (S-N) frame, remains a challenge.

**Methods:**

This study proposes a novel method for predicting the Sella turcica (ST) coordinate from 3D cranial surface models using Principal Component Analysis (PCA) combined with a Feedforward Neural Network (FFNN). The accuracy of this method is compared with the conventional Computed Cranial Focal Point (CCFP) method, which has limitations, especially in cases of asymmetric cranial deformations like plagiocephaly. A data set of 153 CT scans, including 68 craniosynostosis subjects, was used to train and test the PCA-FFNN model.

**Results:**

The results demonstrate that the PCA-FFNN approach outperforms CCFP, achieving significantly lower deviations in ST coordinate predictions (3.61 vs. 8.38 mm, *P*<0.001), particularly along the *y*-axes and *z*-axes. In addition, mesh realignment within the S-N reference frame showed improved accuracy with the PCA-FFNN method, evidenced by lower mean deviations and reduced dispersion in distance maps.

**Conclusions:**

These findings highlight the potential of the PCA-FFNN approach to provide a more reliable, noninvasive solution for cranial assessment, improving craniosynostosis follow-up and enhancing clinical outcomes.

Craniosynostosis, a condition characterized by the premature fusion of one or more cranial sutures, poses significant challenges for patients. This cranial malformation can lead to facial asymmetry, increased intracranial pressure, visual impairment, and impaired neurological development.^1–3^ The prevalence of isolated, nonsyndromic craniosynostosis ranges from 3.1 to 6.4 per 10.000 births, with scaphocephaly, trigonocephaly, and anterior plagiocephaly being the most frequently encountered variants.^4,5^

Early diagnosis and intervention are crucial, as they expand therapeutic options and contribute to optimal surgical outcomes. Specifically, early detection enables the use of the less invasive endoscopic-assisted craniosynostosis surgery (EACS) instead of open reconstruction, reducing surgical burden and recovery time.^6–9^ Traditionally, craniofacial morphologic assessment has relied on radiographic techniques such as computed tomography (CT), which exposes the infant to ionizing radiation.^10,11^ However, the advent of 3D stereophotogrammetry has introduced a radiation-free alternative for evaluation of the cranial shape.^12–15^ This noninvasive approach offers possibilities for accurate postoperative patient follow-up and comparison with a large database with healthy references (ie, noncraniosynostosis).^16^

For such follow-up and comparison, consistent and precise alignment of 3D photographs within a standardized reference frame is vital. A commonly used reference frame for CT scan alignment is the Sella–turcica–Nasion (S-N) frame.^11,13^ However, this frame relies on fixed distances, making it unsuitable for superimposing 3D surface data in growing individuals. To address this limitation, the existing reference frame was adapted to enable the superimposition of 3D soft-tissue images in developing patients. The adapted reference frame is established by annotating 10 cephalometric landmarks, including the endocanthion, exocanthion, nasion, subnasale, supraaurale, and pretragion. A key landmark in the traditional S-N frame, the Sella turcica (ST)—defined as the center of the Sella turcica between the anterior and posterior clinoid processes—cannot be directly identified in 3D photographs. Instead, its position is approximated using the computed cranial focal point (CCFP) combined with a CCFP offset value.^13,17–19^ The adapted S-N reference frame, along with the CCFP point and CCFP offset value, is integrated into the clinical craniosynostosis follow-up protocol at Radboudumc.^18^

Nevertheless, the accuracy of the CCFP-based approximation is compromised in cases of asymmetric cranial deformations, such as anterior plagiocephaly, and the offset value is influenced by variables including age, sex, and craniosynostosis subtype. Consequently, orienting the 3D photograph within the S-N reference frame becomes challenging. Moreover, since the CCFP method is already implemented in standard care during consultation hours with patients, parents, and clinicians, adopting an improved approach is imperative to ensure both enhanced accuracy and seamless clinical integration.

Although several studies have utilized deep learning—employing either convolutional neural networks (CNNs) or U-Nets—to automatically segment the ST from 3D volumetric DICOM data,^20–23^ no research to date has addressed the automatic, machine learning-based detection of the single 3D ST coordinate from a 3D surface model. To address this gap, this study introduces a novel approach combining principal component analysis (PCA) and a feedforward neural network (FFNN) to accurately predict the ST coordinate from 3D cranial surface models. PCA is a statistical technique that simplifies complex 3D data by identifying the most important patterns and reducing unnecessary variations.^24,25^ In this study, PCA helps standardize and align 3D cranial shapes, ensuring consistency before further analysis. Once the data is structured, an FFNN—a type of artificial neural network designed to recognize patterns and make predictions—is trained to estimate the ST coordinate based on the 3D surface model.^26^ For clinicians, this approach offers significant advantages in the consultation room. By improving the accuracy of ST prediction and the alignment of 3D photographs, this method could enable more precise cranial shape assessments over time. This could potentially improve postoperative follow-up, and allow for better communication with parents by providing clear, standardized visualizations of their child’s cranial development. Furthermore, since the CCFP method is already integrated into clinical practice, transitioning to this improved approach ensures both greater accuracy and seamless implementation without disrupting standard workflows.

## METHODS

### Data Acquisition

As the ST is not directly identifiable in 3D photographs, this study utilizes both hard-tissue and soft-tissue information from CT scans to develop a method for extracting the location of the ST and the craniofacial surface, respectively. The soft-tissue data is used to simulate a 3D photograph of the skull. This approach has been previously evaluated, confirming its accuracy in matching 3D stereo photographs with skin surfaces derived from cone-beam CT scans, and has been effectively applied in prior research.^17,27^

A total of 153 CT scans were retrospectively collected, with a mean age of 7.7 months (range: 0.1–46 mo). Within this data set, 85 healthy subjects and 68 subjects with craniosynostosis were identified. Among the 68 craniosynostosis subjects, 22 were diagnosed with trigonocephaly, 25 with scaphocephaly, and 21 with plagiocephaly. All craniosynostosis patients were treated at the Radboud University Medical Center (Radboudumc) and diagnosed with isolated, nonsyndromic premature suture closure, as confirmed by CT. Supplemental Table 1, Supplemental Digital Content 1, http://links.lww.com/SCS/I27 provides an overview of the subject characteristics. Data collection for this study was approved by the medical ethical review board of the Radboudumc, the Netherlands (no. 2020-6128); the study was also reviewed by the ethics committee (Commissie Mensgebonden Onderzoek regio Arnhem-Nijmegen, the Netherlands), and informed consent was waived by the ethics committee.

### Data Preprocessing

**FIGURE 1:**
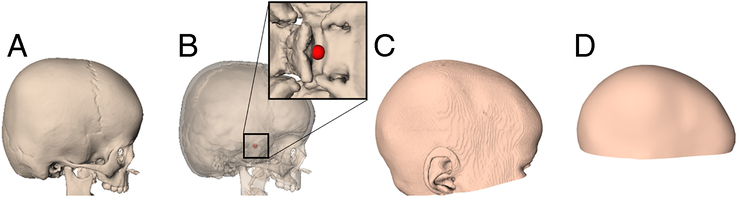
Preprocessing overview. (A) Hard-tissue segmentation. (B) Sella Turcica annotation. (C) Soft-tissue segmentation. (D) Raycasting for 3D cranial surface extraction.

To reconstruct the cranial surfaces, that simulate 3D photographs, from the CT scans, soft-tissue thresholding was applied using 3DMedX software (v1.2.29.0, 3D Lab Radboudumc, Nijmegen, The Netherlands; https://3dmedx.nl). A standard soft tissue preset was selected to include Hounsfield unit (HU) values between −600 and 100. After thresholding, the resulting segmentations were manually cropped from the neck region to the cranial apex. Next, the nasion and the left and right supra-aural landmarks were manually annotated; these landmarks were used to horizontally align the cranial surface models. The resulting surface model was then exported as an STL file and loaded into MATLAB (version 2022b, Mathworks Inc., Natick, MA) to remove internal soft-tissue structures. Within MATLAB, a 3D surface mesh was generated using raycasting by positioning a hemi-icosphere, containing 1016 vertices, at the midpoint of the 2 supra-aural landmarks. The resulting cranial surface model, consisting of 1016 vertices in the Cartesian coordinate system, serves as an alternative to a 3D photograph. Next, a hard-tissue segmentation (thresholding range: 276 HU–1500 HU) was performed for each CT scan using 3DMedX. Subsequently, the ST coordinate was manually annotated on the segmented bony structure, serving as the ground truth (ie, true ST). The ST coordinates were also determined by a second observer to assess interobserver variability by calculating the Euclidean distances between the annotated coordinates. The result of this preprocessing step is a soft-tissue-derived cranial surface model labeled with a hard-tissue-based ST coordinate. An overview of the preprocessing workflow is shown in Figure 1.

### PCA-FFNN Solution

The proposed solution, which predicts the ST coordinates based on the input of a cranial surface model, combines principal component analysis (PCA) and a feedforward neural network (FFNN). The predicted ST coordinate facilitates the orientation of the 3D mesh within the S-N reference frame.

The input data is processed using PCA to transform and reduce the dimensionality of the vertex data to a set of 32 principal components. From these components, the first 10 features containing the highest individual variances were selected, accounting for 98.91% of the total variance in the input data.

After dimensionality reduction, the extracted features serve as the input for the FFNN. In addition, an extra 4-feature vector containing information about the craniosynostosis classification (ie, noncraniosynostosis, plagiocephaly, trigonocephaly, and scaphocephaly) was added. This addition results in an input vector of size 14 (10 features plus 4 classification indicators). The FFNN architecture begins with an initial layer with 8 neurons, followed by a rectified linear unit (ReLU) activation layer and a dropout layer with a dropout rate of 0.5 to prevent overfitting. Subsequently, identity mapping is applied through 3 consecutive layers with 8 neurons each, followed by a final dropout layer with a dropout rate of 0.1. Finally, a linear layer maps the 8-neuron output to the desired output, a 1×3 array representing the ST coordinate. The network was trained using the Adam optimizer, with a learning rate empirically set to 1×10^−3^ and a decay rate of 0.5 every 100 epochs. The batch size was set to 1. Model implementation was performed using PyTorch (v1.12.1) on a machine with an NVIDIA Titan V 12 GB GPU. The model was trained for a total of 400 epochs using a 5-fold cross-validation approach. For the loss function, the mean squared error (MSE) between the predicted and ground truth ST coordinates was computed. Subsequent analyses were conducted on the test set of each fold.

### Performance Analysis

The accuracy of the proposed framework was validated using a 2-fold analysis. First, the accuracy of the predicted ST was evaluated by comparing the predicted ST coordinate to the true ST obtained from the hard-tissue CT scan and to the ST determined by the CCFP method. Differences were quantified using the Euclidean distance, and evaluation was performed separately for each craniosynostosis subtype. Statistical comparisons were performed using IBM SPSS Statistics, Version 28 (IBM Corp, Armonk, NY). Following normality assessment within each group with a Shapiro-Wilks test, a paired *T* test was conducted to compare the ST approximation with a significance level of 0.05; if normality was not met, a Wilcoxon signed-rank test was performed.

**FIGURE 2:**
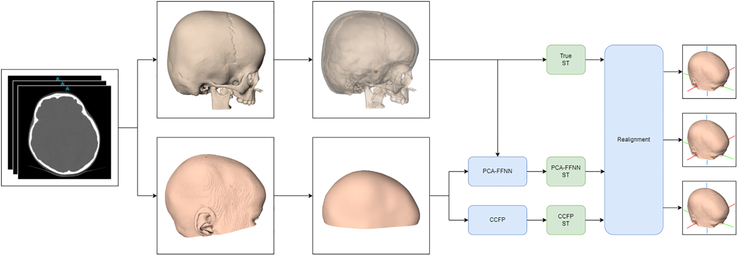
Overview of the proposed method. First, hard- and soft-tissue segmentations are derived. Then the ST is annotated using the hard-tissue segmentation, while a cranial surface is extracted using raycasting. Next, the ST position is predicted using both the CCFP method and the PCA-FFNN approach. Lastly, the 3D cranial surface meshes are realigned.

Second, the orientation of the 3D meshes within the S-N reference frame was evaluated using distance maps representing the distance between two 3D meshes, created with 3DMedX. For the PCA-FFNN method, meshes were reoriented into the S-N reference frame using the predicted ST, nasion, and supra-aural landmarks. For the currently implemented CCFP method, meshes were horizontally aligned using the S-N reference frame and the CCFP landmark, derived from manual landmark annotation. Both orientations were compared with the true orientation, which used the true ST and soft-tissue landmarks (nasion and supra-aural) for S-N orientation. Comparison was done by means of a distance map, of which the fifth percentile, the 95th percentile, and mean deviations were extracted. An overview of the proposed framework is shown in Figure 2.

## RESULTS

The ground truth ST coordinates were manually annotated on the hard-tissue segmented 3D meshes by 2 observers, resulting in an interobserver variability of 1.2 mm. The accuracy of the predicted ST coordinate was evaluated by comparing it to the ground truth, and similarly, the CCFP-derived ST coordinate was compared with the ground truth. The statistical differences between the 2 methods were assessed using *P*-values. Supplemental Table 2, Supplemental Digital Content 1, http://links.lww.com/SCS/I27 shows that the PCA-FFNN approach leads to a significantly lower deviation on the *y*-axis (–3.49 mm, *P*<0.001) and *z*-axis (–2.79 mm, *P*<0.001). In addition, the Euclidean distance between the PCA-FFNN predicted ST and the ground truth (3.61 mm) was significantly lower by 4.77 mm (*P*<0.001) than that between the CCFP predicted ST and the ground truth (8.38 mm). No significant difference in *x*-axis deviation was observed (–0.17 mm, *P*=0.420). When examining subtypes individually, significant differences in accuracy were found for healthy subjects, trigonocephaly, and scaphocephaly. Although the PCA-FFNN approach demonstrated improved landmark accuracy for plagiocephaly, this difference was not statistically significant.

**FIGURE 3:**
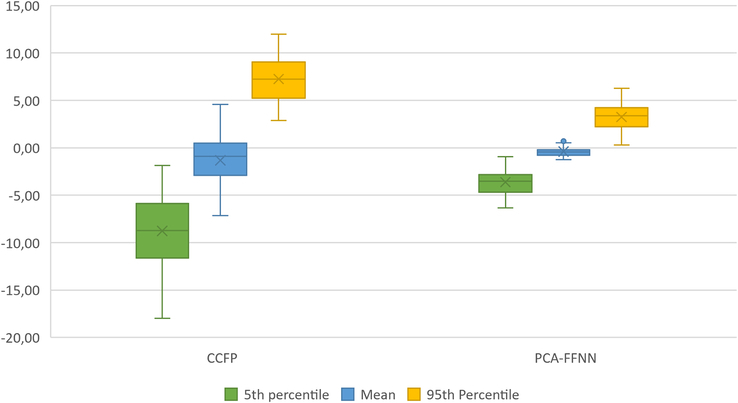
3D distance map evaluation of S-N orientation. Both the CCFP and PCA-FFNN-based orientations are compared with the true ST-based orientation.

In addition to assessing the accuracy of the ST coordinate predictions, the 3 ST coordinates (PCA-FFNN, CCFP, and ground truth) were used to position the 3D cranial surface meshes within the S-N reference frame. Distance maps were generated for each mesh orientation by calculating the differences between the PCA-FFNN predicted ST-oriented mesh and the true ST-oriented mesh, as well as between the CCFP ST-oriented mesh and the true ST-oriented mesh. Figure 3 shows the error distribution for both the PCA-FFNN and CCFP-based orientations for all subtypes combined. After introducing the PCA-FFNN approach, the fifth percentile deviation improved from –8.16 mm to –4.48 mm (*P*<0.001), the 95th percentile deviation from 8.15 mm to 4.38 mm (*P*<0.001), and the mean deviation from –0.79 mm to –0.11 mm (*P*<0.001).

## DISCUSSION

Accurate prediction of the ST coordinate on 3D photographs is crucial for various clinical applications in craniosynostosis management. Previous studies have demonstrated that the ST can be approximated on 3D photographs by calculating the CCFP; however, the CCFP method exhibits limitations when applied to asymmetric cranial deformations such as plagiocephaly. Therefore, this study proposes a novel approach that combines PCA and FFNN to predict the exact ST coordinate from a 3D cranial surface input. The accuracy of both the ST coordinate prediction and the subsequent alignment within the S-N reference frame was evaluated.

The results indicate that the overall accuracy of the PCA-FFNN approach was 3.61±2.03 mm, significantly outperforming the CCFP method, which achieved an accuracy of 8.38±5.21 mm. The most notable improvement in accuracy was observed along the *y*-axis (anteroposterior direction), while no significant improvement was detected along the *x*-axis (mediolateral direction). Subtype analysis revealed a significant increase in accuracy for healthy subjects, as well as for patients with trigonocephaly and scaphocephaly. Although an improvement was observed for plagiocephaly, it did not reach statistical significance, possibly due to the lateral asymmetry inherent to plagiocephaly combined with the lack of significant enhancement in the mediolateral direction.

To our knowledge, no previous research has addressed the prediction of the ST position from 3D surface data using a machine learning-based approach. Several studies have been conducted to automatically segment the ST from CBCT scans;^20,21,23^ however, these approaches rely on volumetric CBCT data for ST segmentation, whereas the method proposed in this study requires only a 3D cranial surface input. Given the anatomic dimensions of the ST (ranging from 4 to 12 mm by 5 to 16 mm) and an interobserver variability of 1.2 mm, the achieved average accuracy of 3.61 mm appears promising.^28^ Furthermore, the alignment of 3D cranial surface models was significantly more accurate using the PCA-FFNN approach compared with the CCFP method, which is consistent with the improved ST prediction accuracy observed. Figure 3 illustrates that the PCA-FFNN approach resulted in decreased overall dispersion. In addition, all boxplot values are located closer to zero, compared with the CCFP-based orientation method. These findings highlight the potential of the PCA-FFNN method for more accurate alignment of 3D cranial surface models and improved craniosynostosis follow-up.

### Limitations

A limitation of this study is the relatively small data set (n=150), which may increase the risk of overfitting the FFNN. Nonetheless, precautions such as dimensionality reduction through PCA, data augmentation, and early stopping were implemented to mitigate this risk. Furthermore, the distribution between healthy subjects and craniosynostosis patients was unequal, particularly after subdividing into subtypes. Expanding the craniosynostosis database could further enhance both the accuracy of ST prediction and the subsequent alignment of 3D cranial meshes.

Another major limitation is the absence of time-matched CT scans and 3D photographs. The reliance on CT-derived soft-tissue segmentations, rather than actual 3D photographs, restricts the immediate clinical applicability of the PCA-FFNN approach. Since the FFNN was trained on CT-derived 3D meshes, its performance on true 3D photographs remains uncertain. Future work should apply the proposed workflow to 3D photographs and expand the database to include time-matched CT scans and 3D photographs. While the use of soft-tissue segmentation introduces certain limitations, it also offers the advantage of an exact correlation between the cranial surface and the annotated ST location. Future research should thus focus on expanding or replacing the database with time-matched CT scans and 3D photographs, as well as investigating the precise accuracy of ST prediction and mesh alignment when using 3D photographs. Finally, it would be valuable to explore whether the PCA-FFNN method could be extended to automatically realign the 3D mesh without requiring additional manually annotated craniofacial landmarks.

## CONCLUSIONS

In conclusion, this paper introduces a novel approach for predicting the Sella turcica and aligning 3D cranial surface meshes within the S-N reference frame. To our knowledge, this is the first study to employ a neural network for predicting a 3D landmark that lies beyond the surface mesh. Our findings demonstrate that the PCA-FFNN approach outperforms the conventional CCFP method in both ST prediction and mesh realignment.

### Future Perspectives

Integrating the PCA-FFNN method into clinical practice enhances craniosynostosis diagnosis and postoperative monitoring while ensuring a seamless transition from the existing CCFP approach at Radboudumc. This improves diagnostic accuracy without disrupting workflows. By incorporating PCA-FFNN into stereophotogrammetry-based assessments, clinicians can achieve more precise 3D cranial alignments in real-time, reducing manual landmark annotation and interobserver variability. The method’s nonreliance on ionizing radiation supports the trend of minimizing CT scans in pediatric patients. Enhanced accuracy aids clearer communication with parents, providing detailed 3D visualizations of cranial changes to illustrate treatment progress and surgical outcomes. Long-term monitoring and comparisons with normative databases help detect subtle asymmetries, optimize treatment timing, and refine intervention strategies. Future research should validate this approach in larger cohorts and develop automated software tools for clinical integration. Ultimately, adopting PCA-FFNN offers a standardized, reliable, and noninvasive method for craniosynostosis assessment.

## Supplementary Material
