# Supplementary material for: Automated Sella-Turcica Annotation and Mesh Alignment of 3D Stereophotographs for Craniosynostosis Patients Using a PCA-FFNN Based Approach
Source: J Craniofac Surg. 2025 Jun 27;37(5):919–23. doi: 10.1097/SCS.0000000000011623 (PMC13117562; doi:10.1097/SCS.0000000000011623)
Supplement: Supplementary file 1 [file scs-37-0919-s001.docx]

**Supplemental Digital Content**

Supplemental Table 1: Database characteristics.

| **Characteristics** | **Number** | **Age (months)** | **Gender (m/f)** |
| --- | --- | --- | --- |
| Non-craniosynostosis | 85 | 18.22 (0.1-46) | 45/40 |
| Craniosynostosis | 68 |  |  |
| *Scaphocephaly* | *25* | 4.90 (2.6-12.7) | 20/5 |
| *Trigonocephaly* | *22* | 4.10 (1.9-10.7) | 16/6 |
| *Plagiocephaly* | *21* | 2.76 (0.1-6) | 5/16 |
| *Total* | *153* | *7.65* | *86/67* |

Supplemental Table 2: Accuracy measurements for both the CCFP and PCA-FFN based ST predictions. A p-value <0.001 shows the presence of significant differences between the two approaches.

|  | **CCFP** | **PCA-FFNN** | **PCA-FFNN < CCFP** |
| --- | --- | --- | --- |
| **Comparison** | **Abs. mean ±SD (mm)** | **Abs. mean ±SD (mm)** | **p-value** |
| X-axis | 1.42 ±1.15 | 1.25 ±0.98 | p = 0.420^a^ |
| Y-axis | 5.40 ±2.98 | 1.91 ±1.80 | p < 0.001^a^ |
| Z-axis | 4.87 ±2.58 | 2.08 ±1.83 | p = 0.005^a^ |
| Euclidean – all data | 8.38 ±5.41 | 3.61 ±2.03 | p < 0.001^a^ |
| Euclidean – healthy | 8.71 ±6.65 | 3.57 ±1.63 | p < 0.001 |
| Euclidean – plagiocephaly | 5.08 ±2.28 | 4.06 ±2.22 | p = 0.079 |
| Euclidean – trigonocephaly | 9.07 ±2.63 | 3.30 ±2.16 | p < 0.001 |
| Euclidean – scaphocephaly | 9.48 ±2.84 | 3.66±2.74 | p < 0.001 |

^a^Data was not normally distributed so a Sign Test was performed.
